# The diagnostic accuracy of circulating tumor DNA for the detection of EGFR-T790M mutation in NSCLC: a systematic review and meta-analysis

**DOI:** 10.1038/s41598-018-30780-4

**Published:** 2018-09-06

**Authors:** Francesco Passiglia, Sergio Rizzo, Massimo Di Maio, Antonio Galvano, Giuseppe Badalamenti, Angela Listì, Leonardo Gulotta, Marta Castiglia, Fabio Fulfaro, Viviana Bazan, Antonio Russo

**Affiliations:** 1 Department of Surgical, Oncological and Oral Sciences, Section of Medical Oncology, University of Palermo, Palermo, Italy; 2 Division of Medical Oncology, Umberto I “Ordine Mauriziano” Hospital, Via Magellano 1, Turin, 10028 Italy; 3 Department of Oncology, University of Turin, Turin, Italy; 4 Department of Surgical, Oncological and Stomatological Disciplines, University of Palermo, Palermo, Italy

## Abstract

This pooled analysis aims at evaluating the diagnostic accuracy of circulating tumor (ct) DNA for the detection of EGFR-T790M mutation in NSCLC patients who progressed after EGFR-TKIs. Data from all published studies, reporting both sensitivity and specificity of plasma-based EGFR-T790M mutation testing by ctDNA were collected by searching in PubMed, Cochrane Library, American Society of Clinical Oncology, European Society of Medical Oncology and World Conference of Lung Cancer meeting proceedings. A total of twenty-one studies, with 1639 patients, were eligible. The pooled sensitivity of ctDNA analysis was 0.67 (95% CI: 0.64–0.70) and the pooled specificity was 0.80 (95% CI: 0.77–0.83). The pooled positive predictive value (PPV) was 0.85 (95% CI: 0.82–0.87) and the pooled negative predictive value (NPV) was 0.60 (95% CI: 0.56–0.63). The positive likelihood ratio (PLR) and negative likelihood ratio (NLR) were 2.67 (95% CI: 1.86–3.82) and 0.46 (95% CI: 0.38–0.54), respectively. The pooled diagnostic odds ratio (DOR) was 7.27 (4.39–12.05) and the area under the curve (AUC) of the summary receiver operating characteristics (sROC) curve was 0.77. The ctDNA analysis represents a promising, non-invasive approach to detect and monitor the T790M mutation status in NSCLC patients. Development of standardized methodologies and clinical validation are recommended.

## Introduction

The identification of epidermal growth factor receptor (EGFR) activating mutations as targetable oncogene drivers produced a paradigm shift in the treatment of lung cancer, leading to a personalized approach according to the patients’ genomic profile. Several randomized phase III trials^1–8^ demonstrated a significant survival benefit of EGFR-tyrosine kinase inhibitors (TKIs) over platinum-chemotherapy for naïve patients with advanced non-small cell lung cancer (NSCLC) carrying exon 19 deletion or exon 21 L858R point mutation. These data led to the clinical approval of first and second-generation TKIs as new standard upfront therapy in about 40% of Asian and 12% of Caucasian “EGFR-positive” NSCLC patients^9^. Unfortunately, although the great benefit obtained with the EGFR-TKIs, cancer cells inevitably develop resistance, commonly within a median time on treatment of 10–12 months, leading to treatment discontinuation as consequence of disease progression. Among the multiple molecular mechanisms responsible for the occurrence of acquired resistance, the secondary T790M point mutation in exon 20 has been identified in about 50–60% of tumor samples after TKI treatment, emerging as the leading cause of disease progression^10,11^. Recently, the scientific community of oncologists has celebrated the advent of the first new-generation EGFR-TKI osimertinib in clinical setting. This compound, differently from the other drugs, is able to selectively target and inhibit not only EGFR activating mutations, but also the resistant T790M mutation^12^. The randomized phase III AURA trial showed a significant survival benefit along with a more tolerable safety profile in favour of osimertinib over platinum-chemotherapy in patients with advanced NSCLC who progressed to prior EGFR-TKIs and were T790M-positive^13^. After the clinical approval of osimertinib as new standard of care in T790M-positive NSCLC patients who received prior EGFR-TKIs, re-biopsy at progression became mandatory, in order to evaluate T790M status and ultimately personalize second-line treatment. Even if tumor tissue biopsy has been considered the gold-standard practice for a long time, however it is associated with several limitations, including the invasiveness of procedures, low patients’ compliance, lengthy turnaround time and intra-tumor heterogeneity. Furthermore, it often produces inadequate material for mutational testing. The detection of EGFR mutations by circulating tumor (ct) DNA has recently emerged as a valid and non-invasive alternative approach, overall showing a high concordance with the standard tissue genotyping^14–16^. Oxnard *et al*. first showed that plasma genotyping by droplet digital PCR (ddPCR) was associated with 70% of sensitivity and 69% of specificity as compared to standard tumor tissue analysis of EGFR-T790M mutation in about 60 NSCLC patients with acquired resistance to EGFR-TKIs^17^. After that, several other studies investigated the diagnostic accuracy of ctDNA in NSCLC patients who progressed to prior EGFR-TKIs, showing a very wide range of concordance rate with the tumor tissue analysis^18–35^. Furthermore the low number of patients included in the majority of such studies has limited both the statistical significance and the scientific reliability of results. Thus to date the diagnostic accuracy of plasma ctDNA analysis for the detection of EGFR-T790M remains still unclear. The aim of this pooled analysis is to combine and analyze simultaneously all the studies comparing ctDNA to tumor tissues based T790M-genotyping in order to provide a more precise estimation of the diagnostic accuracy of ctDNA analysis in patients with EGFR-mutant advanced NSCLC.

## Results

### Characteristics of eligible studies

**Figure 1:**
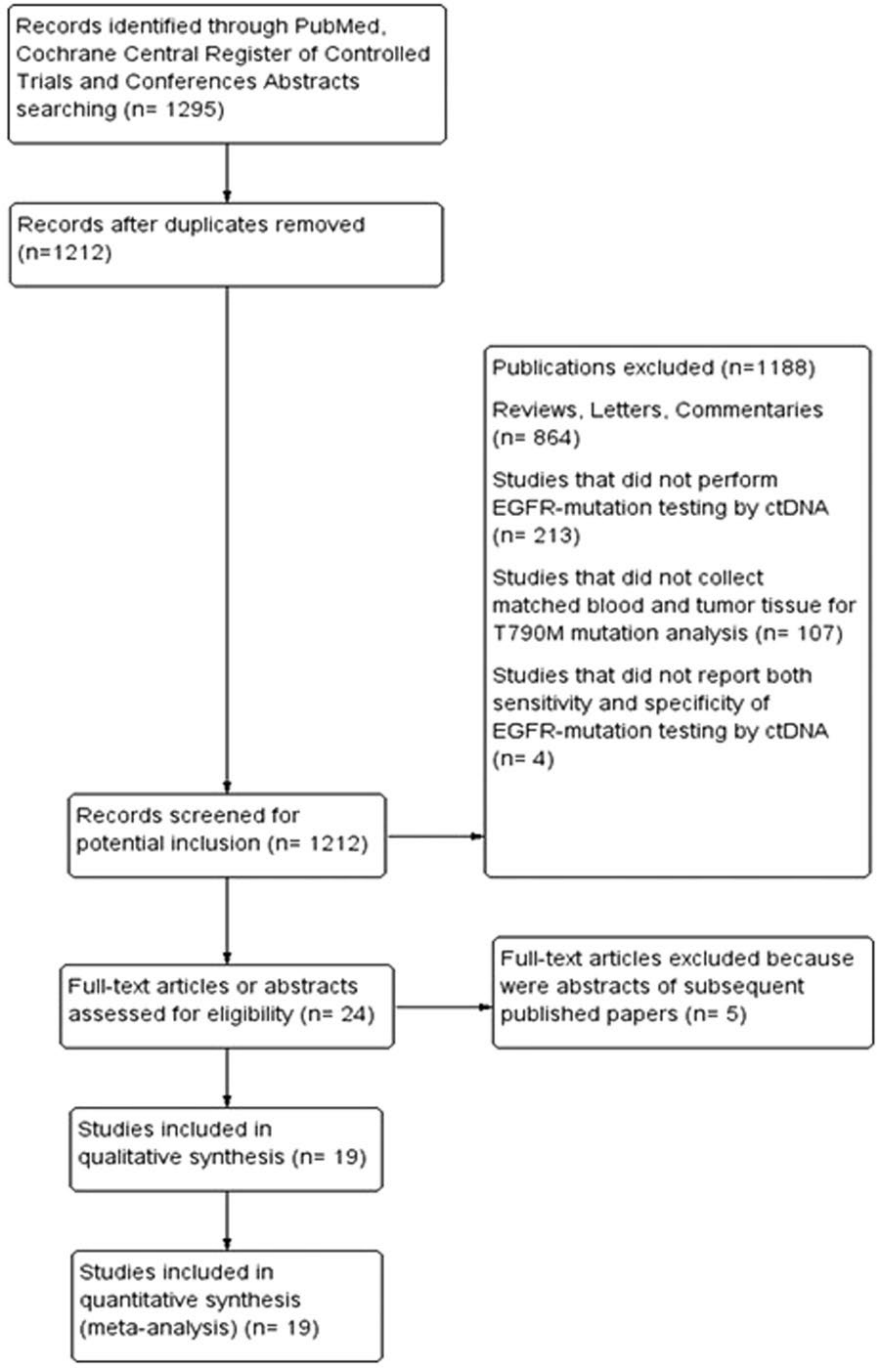
Flow-chart of Trials Selection.

The search of literature updated in March 2018, identified a total of 1295 records. Among these only nineteen studies met our inclusion criteria and were included in the pooled-analysis (Fig. 1). The studies by Karlovic^20^ and Thress^19^ performed the EGFR-T790M mutation analysis by both real time-PCR (RT-PCR) and digital-PCR (dPCR) and the data were reported as two independent studies. Thus overall twenty-one eligible studies (1639 patients) were included in the final analysis. All these studies collected matched blood and tumor tissue from patients with histologically-confirmed diagnosis of advanced NSCLC who progressed to prior EGFR-TKI. The dPCR was the most frequent technology used to detect EGFR-T790M in 12/21 studies, followed by RT-PCR used in 6/21 studies, and next generation sequencing (NGS) used in 3/21 studies. The studies used different thresholds of positivity, including mutant allele concentration (copies/ml), mutant allele fraction (%), and number of droplets, whereas the threshold of positivity was not reported in 8 of included trials. The sample sizes of the analyzed population ranged from 10 to 543 patients. All the studies analyzed both sensitivity and specificity of ctDNA analysis for the detection of EGFR-T790M mutation as compared to the gold standard tumor tissue. The sensitivity of ctDNA analysis ranged from 40% to 93% and the specificity from 18% to 100% across the different studies. The characteristics of the included trials are described in Table 1.

**Table 1:** Characteristics of Trials Included in the Meta-Analysis.

Study (reference)	Number of patients	Assay	Sensitivity n. (%)	Specificity n. (%)	PPV n. (%)	NPV n. (%)
Ishii *et al*.^18^	18	Droplet dPCR	9/11 (81.8)	6/7 (85.7)	9/10 (90)	6/8 (75)
Thress *et al*.^19^	65	RT-PCR (cobas) BEAMing dPCR	30/41 (73) 33/41 (81)	16/24 (67) 14/24 (58)	30/38 (79) 33/43 (76.7)	16/27 (59.3) 14/22 (63.6)
Karlovich *et al*.^20^	95	RT-PCR (cobas) BEAMing dPCR	21/33 (64) 33/45 (73)	61/62 (98) 9/18	21/22 (95.5) 33/42 (78.6)	61/73 (83.6) 9/21 (42.9)
Oxnard *et al*.^17^	216	BEAMing dPCR	111/158 (70.3)	40/58 (69)	111/129 (86)	40/87 (46)
Reckamp *et al*.^21^	105	NGS	38/41 (93)	60/64 (94)	38/42 (90.5)	60/63 (95.2)
Sacher *et al*.^22^	54	Droplet dPCR	27/35 (77)	12/19 (63)	27/34 (79.4)	12/20 (60)
Sundaresan *et al*.^23^	25	RT-PCR (cobas)	6/10 (60)	9/15 (60)	6/12 (50)	9/13 (69.2)
Takahama *et al*.^24^	41	Droplet dPCR	20/31 (65)	7/10 (70)	20/23 (87)	7/18 (38.9)
Paweletz *et al*.^25^	14	NGS	8/10 (80)	2/4 (50)	8/10 (80)	2/4 (50)
Seki *et al*.^26^	10	Droplet dPCR	5/7 (71)	3/3 (100)	5/5 (100)	3/5(60)
Thompson *et al*.^27^	50	NGS	2/4 (50)	40/46 (87)	2/8 (25)	40/42 (95.2)
Suzawa *et al*.^28^	59	Droplet dPCR	9/21 (36)	37/38 (97)	9/10 (90)	37/49 (75.5)
Jenkins *et al*.^29^	543	RT-PCR (cobas)	255/416 (61.4)	100/127 (78.6)	255/282 (90.4)	100/261 (38.3)
Wang *et al*.^30^	16	Droplet dPCR	6/9 (66.7)	5/7 (71.4)	6/8 (75)	5/8 (62.5)
Mellert *et al*.^31^	55	Droplet dPCR	13/15 (87)	40/40 (100)	13/13 (100)	40/42 (95.2)
Kasahara *et al*.^32^	20	Chip-based dPCR	5/7 (71	7/13 (54)	5/11 (45.5)	7/9 (77.8)
Yoshida *et al*.^33^	21	PNA-LNA PCR	4/10 (40)	11/11 (100)	4/4 (100)	11/17 (64.7)
Wu *et al*.^34^	24	RT-PCR	7/17 (41)	5/7 (71)	7/9 (77.8)	5/15 (33.3)
Buder *et al*.^35^	45	Droplet dPCR	28/34 (82)	2/11 (18)	28/37 (75.7)	2/8 (25)

RT-PCR: real-time PCR; dPCR: digital-PCR; NGS: next-generation sequencing; CI: confidence intervals; PPV: positive predictive value; NPV: negative predictive value.

### Diagnostic accuracy analysis

**Figure 2:**
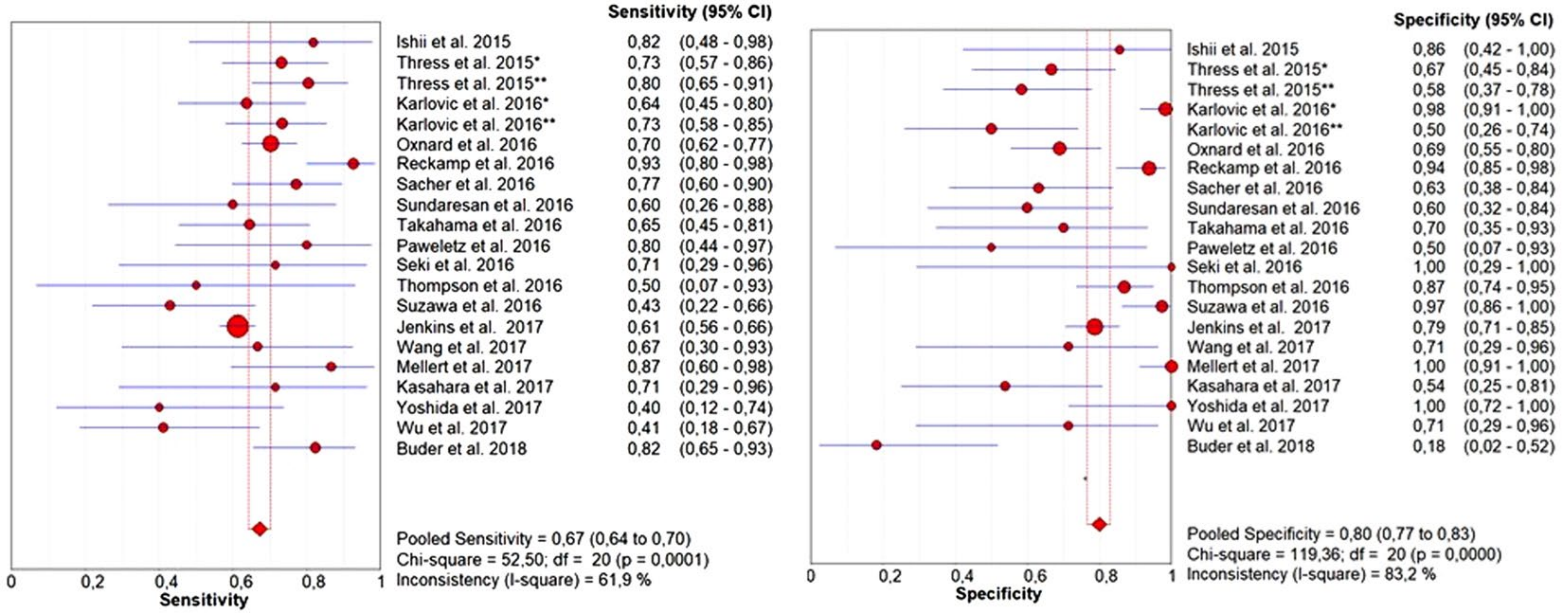
Forest plots of sensitivity and specificity of ctDNA for the detection of EGFR-T790M mutation; *RT-PCR; **dPCR.

**Figure 3:**
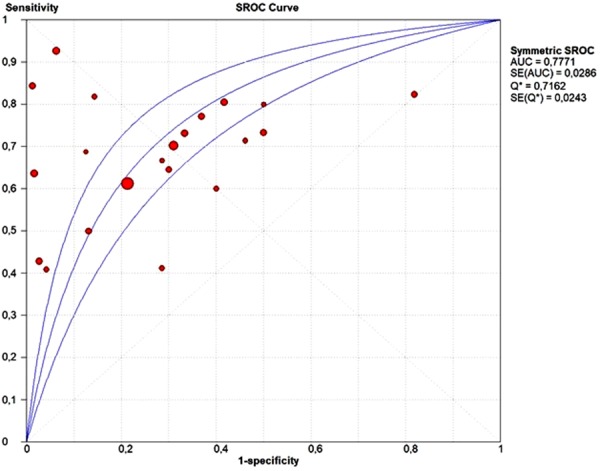
SROC curve of ctDNA for detection of EGFR-T790M mutation.

**Figure 4:**
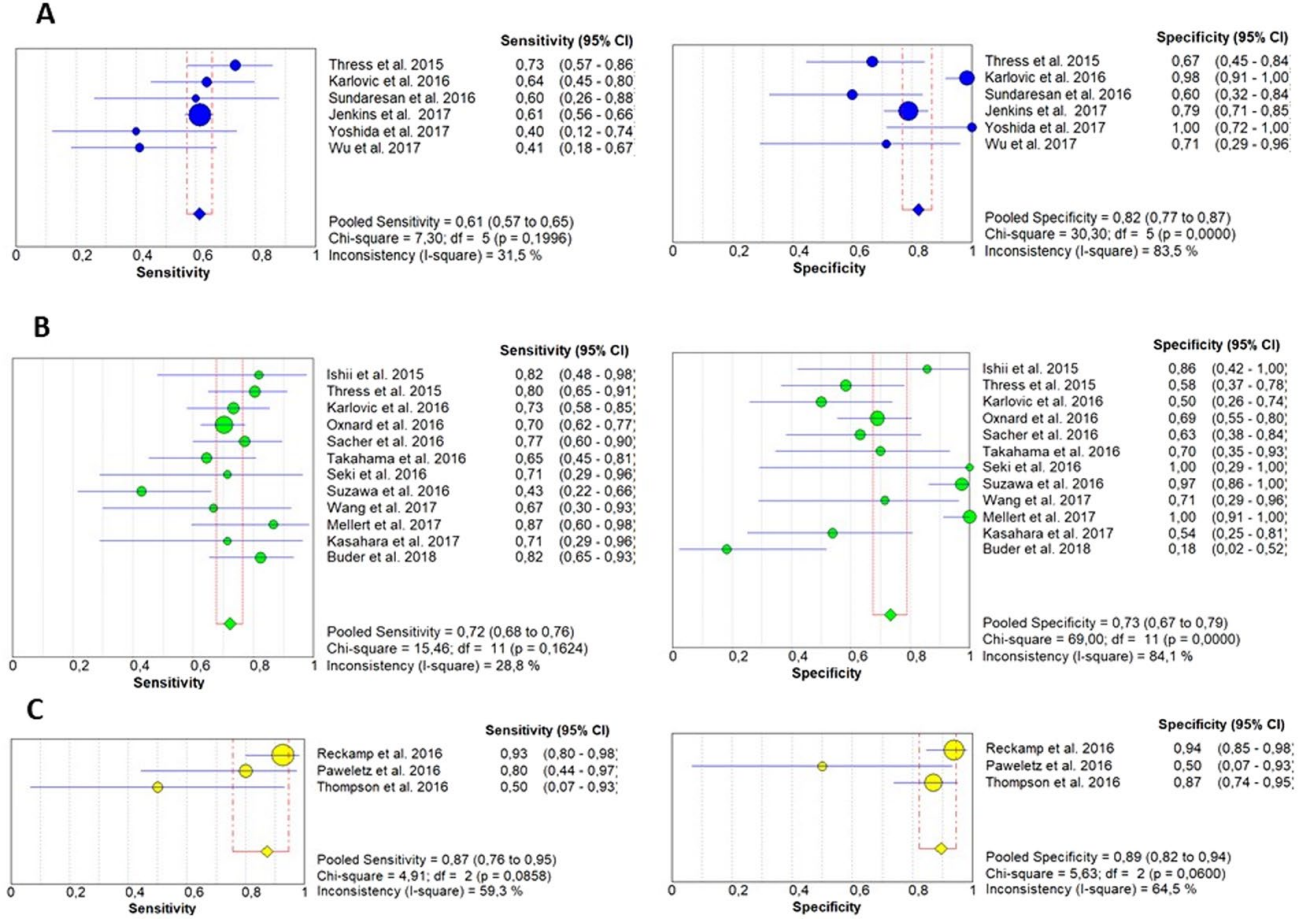
Forest plots of sensitivity and specificity of ctDNA for the detection of EGFR-T790M mutation according to the different diagnostic methods: (**A**) (RT-PCR); (**B**) (dPCR); (**C**) (NGS).

**Figure 5:**
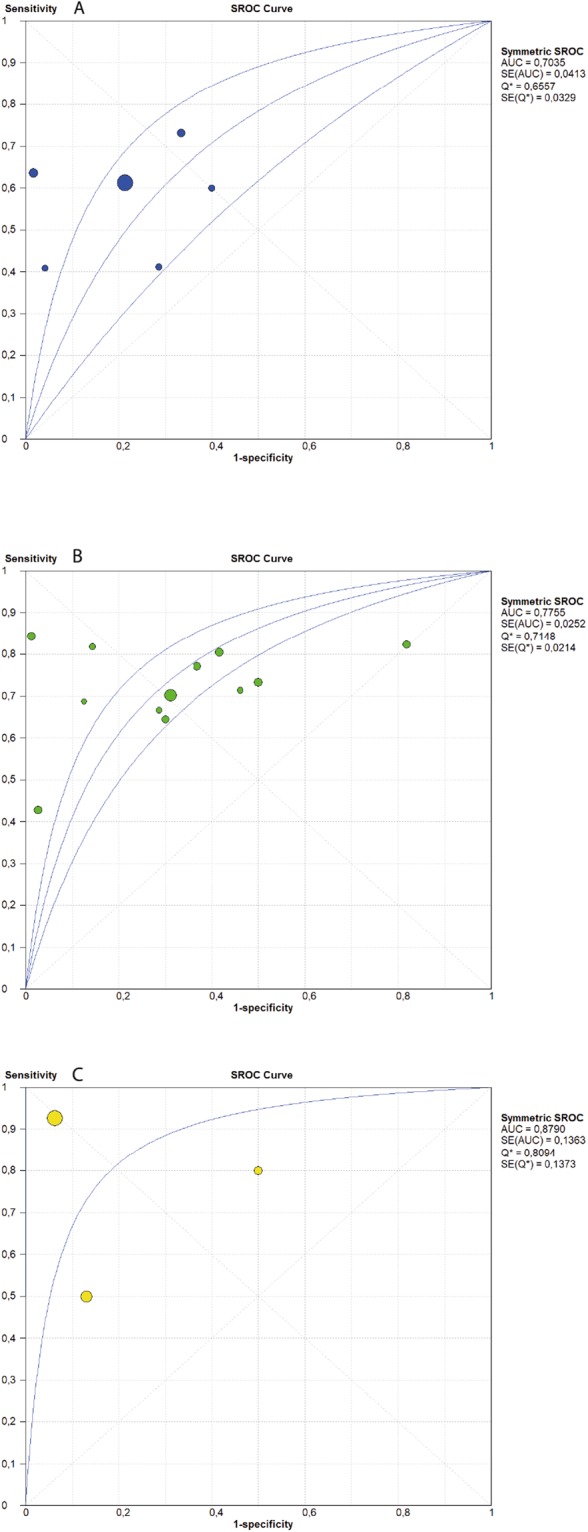
SROC curve of ctDNA for detection of EGFR-T790M mutation according to the different diagnostic methods: (**A**) (RT-PCR); (**B**) (dPCR); (**C**) (NGS).

The pooled sensitivity of ctDNA was 0.67 (95% CI: 0.64–0.70) and the pooled specificity was 0.80 (95% CI: 0.77–0.83) (Fig. 2). The pooled positive predictive value (PPV) of ctDNA was 0.85 (95% CI: 0.82–0.87) and the pooled negative predictive value (NPV) was 0.60 (95% CI: 0.56–0.63). The positive likelihood ratio (PLR) and negative likelihood ratio (NLR) were 2.67 (95% CI: 1.86–3.82) and 0.46 (95% CI: 0.38–0.54), respectively. The pooled diagnostic odds ratio (DOR) was 7.27 (4.39–12.05) and the area under the curve (AUC) of the summary receiver operating characteristics (sROC) curve was 0.77 (Fig. 3). Subgroup analysis was performed to investigate if the sample size and the different detection methods could significantly influence the diagnostic accuracy of ctDNA analysis and the results are shown in Figs 4 and 5 and reported in Table 2.

**Table 2:** Meta-Analysis Results.

	Sensitivity (95% CI)	Specificity (95% CI)	PLR (95% CI)	NLR (95% CI)	DOR (95% CI)	AUC (95% CI)
All Studies	0.67 (0.64–0.70)	0.80 (0.77–0.83)	2.67 (1.86–3.82)	0.46 (0.38–0.54)	7.27 (4.39–12.05)	0.78
Sample size Large Studies (>60 patients)	0.67 (0.64–0.71)	0.80 (0.75–0.84)	3.01 (1.86–4.89)	0.39 (0.30–0.52)	9.69 (4.36–21.55)	0.82
Sample size Small Studies (<60 patients)	0.67 (0.61–0.74)	0.81 (0.75–0.85)	2.48 (1.46–4.22)	0.53 (0.44–0.65)	5.66 (2.91–10.99)	0.74
Detection Method (RT-PCR)	0.61 (0.57–0.65)	0.82 (0.77–0.87)	2.71 (1.51–4.87)	0.50 (0.42–0.61)	6.28 (2.15–14.58)	0.70
Detection Method (dPCR)	0.72 (0.68–0.76)	0.73 (0.67–0.79)	2.16 (1.42–3.27)	0.45 (0.38–0.54)	5.67 (3.22–9.98)	0.77
Detection Method (NGS)	0.87 (0.76–0.95)	0.89 (0.82–0.94)	4.55 (1.00–20.83)	0.26 (0.06–1.20)	19.53 (1.44–264.76)	0.88

CI: confidence intervals; RT-PCR: real-time PCR; dPCR: digital-PCR; NGS: next generation sequencing; PLR: positive likelihood ratio; NLR: negative likelihood ratio; DOR: diagnostic odds ratio; AUC: area under curve.

### Threshold effect and heterogeneity

Spearman correlation coefficient and *p-*value were calculated to assess the threshold effect by Meta-DiSc meta-analysis software^36^. The Spearman correlation was 0.296 and the *p* value was 0.193 (>0.05), indicating that the threshold effect was not significant. Since a significant heterogeneity between studies caused by non-threshold has been detected, meta-regression was performed to identify the source of heterogeneity. However the results showed that both sample size and detection methods were not associated to heterogeneity.

### Quality assessment of studies and publication bias

**Figure 6:**
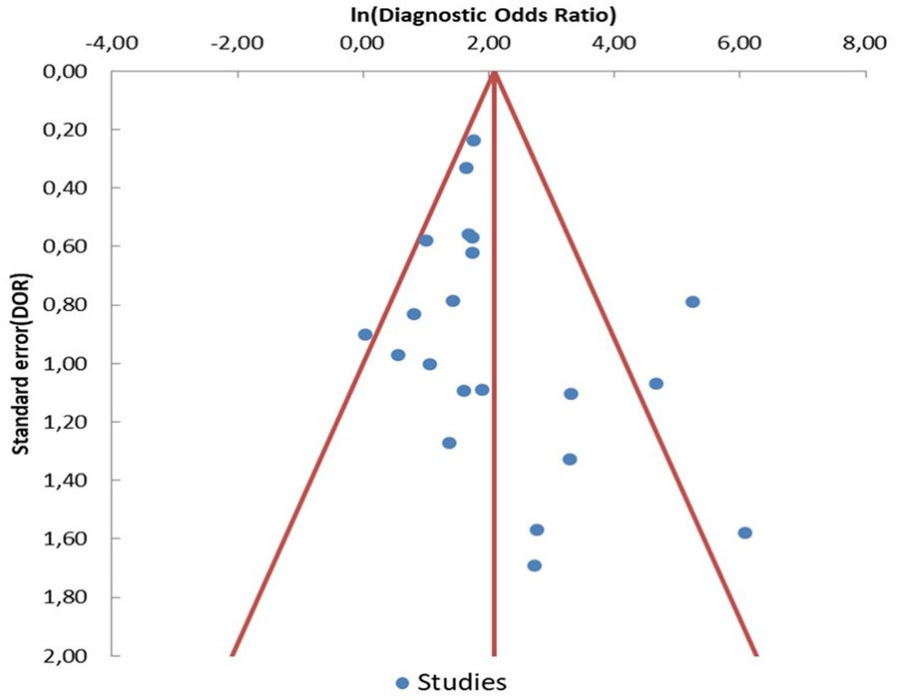
Funnel plot of diagnostic odds ratio (DOR) for ctDNA detection of EGFR-T790M mutation. Each study is represented by one circle- the vertical line represents the pooled effect estimate.

**Figure 7:**
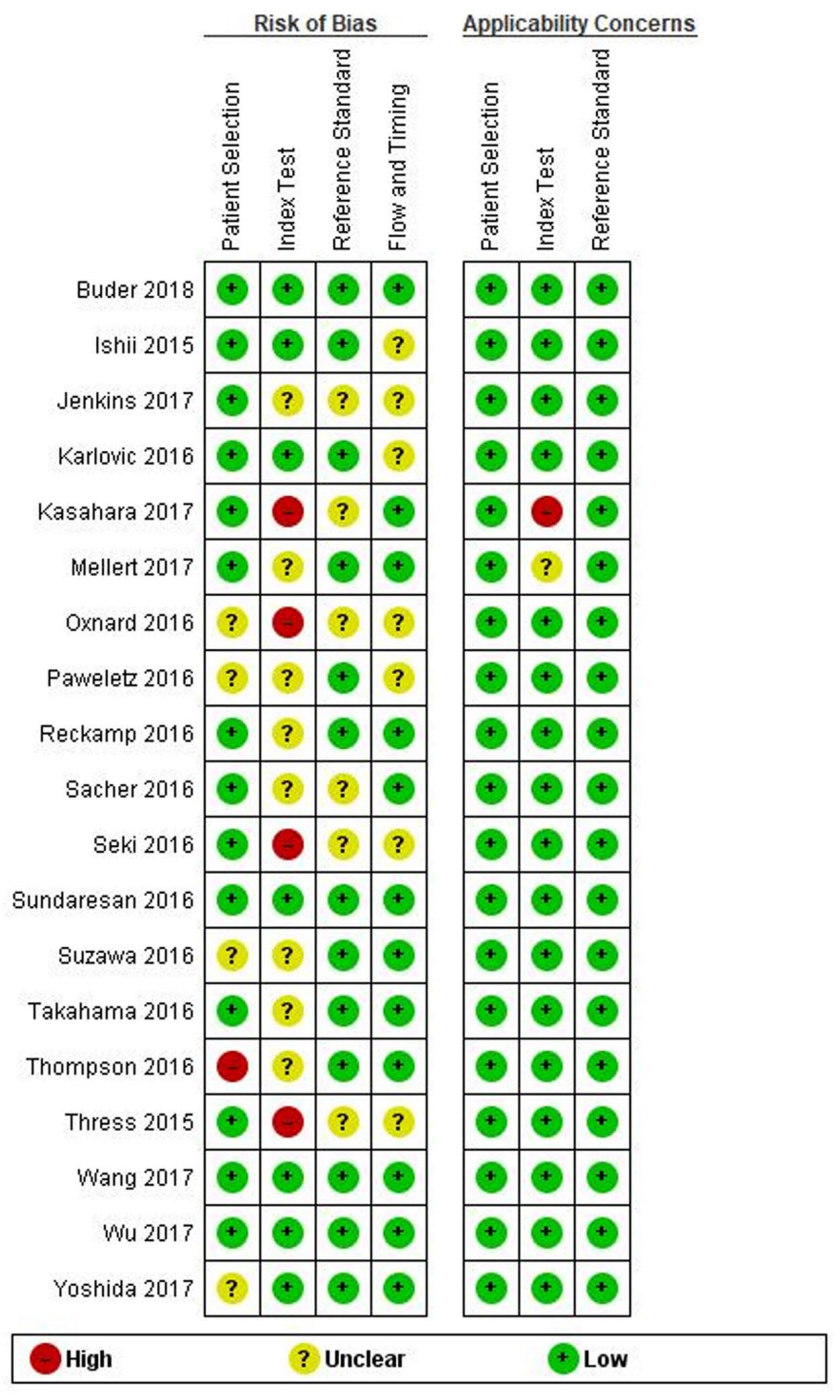
Quality assessment of studies by QUADAS-2.

Egger’s regression test was performed to detect any publication bias. As shown in Fig. 6, no significant publication bias has been found (p = 0.11). The methodological quality of each trial was assessed by QUADAS-2, showing that the overall quality of included studies was good (Fig. 7).

## Discussion

This meta-analysis included twenty-one studies (1639 patients) investigating the diagnostic accuracy of EGFR-T790M mutation testing by ctDNA in patients with advanced NSCLC who progressed to prior EGFR-TKI. The results of this work showed that ctDNA analysis is characterized by a pooled sensitivity of 0.67 and a pooled specificity of 0.80, while pooled PPV and NPV were 0.85 and 0.60, respectively. The ctDNA sensitivity was similar to that reported for the detection of EGFR-activating mutations^14,15^ and it is commonly considered adequate for a cancer screening test. It means that more than 30% of patients with a T790M-positive tissue biopsy will result negative on plasma test. Furthermore, as specifically shown by Jenkins *et al*. the false-negative rate may grow up to 50% in patients with intra-thoracic limited (M1a) disease^29^. For this reason, as recommended by Oxnard *et al*. any case of negative result on ctDNA analysis should be always confirmed by tumor tissue analysis^17^. Conversely, the ctDNA specificity emerged from this analysis was somewhat lower than that observed for EGFR-activating mutations. Suboptimal specificity could be in part explained by the high intra-tumor heterogeneity, increasing the chances of “false negative” results by tumor tissue analysis. Oxnard *et al*. have recently demonstrated that a subgroup of plasma T790M-positive patients receiving osimertinib in the AURA 1 study who were T790M-negative on tissue analysis had favorable clinical outcomes similarly to T790M-positive patients on tumor tissue^17^. Similarly 23/27 patients included in both AURA 1 and 2 studies who were initially detected as T790M-positive on plasma testing and T790M-negative by tumor tissue RT-PCR-based genotyping were confirmed to be T790M-positive by tissue NGS, showing favourable outcomes comparable to T790M-positive population^37^. Interestingly Chabon *et al*. identified additional molecular alterations by CAncer Personalized Profiling by deep Sequencing (CAPP-Seq) ctDNA analysis in 46% of patients who were T790M-positive on tumor tissue, including cMET or HER2 increased gene copy number (GCN), and single nucleotide variations (SNVs) in EGFR, PIK3CA, or Rb1, suggesting that ctDNA analysis allows to more accurately identify the presence of multiple mechanisms of resistance emerging during TKI therapy^38^. Overall these data confirmed that the lower specificity of ctDNA observed in EGFR-TKI-resistant NSCLC patients could be partially related to the higher intra-tumor heterogeneity. Thus ctDNA should be considered as more representative of the overall tumor mutation status, allowing to identify plasma T790M mutations which may be missed on tissue biopsy. It is well known that both AUC and DOR are used as indicators to estimate the global performance of a diagnostic tests. According to the current guidelines^39,40^ the AUC of 0.77 and DOR of 7.27 reported in our analysis are not high enough to suggest an elevated diagnostic accuracy of ctDNA analysis of T790M. Similarly both the positive and the negative likelihood ratios suggested that ctDNA did not accurately detect the presence of T790M mutation in the plasma of NSCLC patients. The heterogeneity of analyzed studies, including different sample size as well as different practices and timing for both tumor and plasma sample collection and testing could have negatively influenced the overall results of this work. A stratified analysis was performed to investigate the impact of specific detection methods on the diagnostic accuracy of ctDNA analysis. As reported in Fig. 4 the results of this analysis showed that dPCR is characterized by higher sensitivity but lower specificity as compared to the standard non-digital PCR technologies. These data are in line with the evidences emerged from two selected trials^19,20^ directly comparing the diagnostic performance of BEAMing dPCR to cobas RT-PCR. Interestingly NGS revealed the highest accuracy to identify plasma T790M as compared to other detection platforms. This is likely due to the very high sensitivity of NGS showing the ability to detect mutations at an allele fraction <1% both in tissue and plasma samples^25^. The use of NGS would likely allow to overcome the two main causes of EGFR mutation status discordance between tissue and plasma analysis, such as the intra-tumor heterogeneity and the low sensitivity of the standard diagnostic techniques. However the low number of studies as well as the small sample sizes of the analyzed population in the NGS subgroup might have biased the results, which need to be cautiously interpreted. Worth of mention is also the wide range of positivity thresholds used to detect T790M in both digital and non-digital PCR studies, which represents a major factor influencing both the sensitivity and specificity of a diagnostic test. Additional studies are urgently needed to standardize and improve technical approach and to identify the minimum biological threshold with clinical relevance to guide treatment decisions in clinical practice. Finally some studies and a recent meta-analysis^41^ confirmed that the ability to identify EGFR-T790M mutation in ctDNA significantly varies by the extent of disease, suggesting that higher T790M rate is related to high ctDNA levels regardless of used diagnostic methods. Thus major efforts are needed to select the ideal subset of patients candidate to liquid biopsy where plasma testing could definitively replace tumor tissue analysis. In conclusion this is the first meta-analysis of the diagnostic performance of cfDNA for the detection of EGFR-T790M mutation status in NSCLC patients who progressed to EGFR-TKIs and represents an attempt to provide guidance for future studies. The development of standardized methodologies for ctDNA analysis and clinical validation in prospective trials with large cohorts of patients should be warranted in the near future.

## Materials and Methods

### Search for clinical trials

We searched for all published studies reporting the sensitivity and the specificity of plasma-based EGFR-T790M mutation testing by ctDNA. We searched for clinical trials using Medline (PubMed), Embase-databases and Cochrane-Library up to March 2018. We used the following search terms: “EGFR”, “Epidermal growth factor receptor”, “T790M” “circulating tumor DNA”, “ctDNA”, “non-small cell lung cancer”, “NSCLC”, “lung cancer”. The search was limited to human studies in the English language. Relevant abstracts from the American Society of Clinical Oncology (ASCO), European Society of Medical Oncology (ESMO), and World Conference on Lung Cancer (WCLC) were included. We also explored the ClinicalTrials.gov website (www.clinicaltrials.gov) to search for unpublished data.

### Selection criteria

According to the aforementioned search, clinical trials were taken into account if they met the following inclusion criteria: 1) patients with histologically-proven diagnosis of advanced NSCLC; 2) patients with radiological confirmed progression disease to EGFR-TKI; 3) studies performing EGFR-T790M mutation testing in matched tumour tissue and plasma samples 4) studies evaluating both the sensitivity and the specificity of EGFR-T790M mutation testing by ctDNA analysis. We excluded ongoing studies in order to minimize the risk of bias. In case of articles or abstracts with multiple follow up reports over time, we selected those reporting the most updated data. We excluded also studies which did not simultaneously report both sensitivity and specificity of EGFR-T790M mutation testing by ctDNA analysis.

### Data extraction

Data extraction and assessment was independently made by two different authors (F.P. and S.R.) and disagreements were solved by discussion with another author (M.D.M.). The following data were collected from eligible studies: first author name, journal and year of publication, study design, study treatment, number of patients, baseline characteristics of patients (i.e. age, gender, stage, etc…), methods used for EGFR-T790M detection in ctDNA, true positive (TP), false negative (FN), true negative (TN), and false positive (FN) rates. When multiple methods were used to detect EGFR-T790M in ctDNA, all were extracted and described. The meta-analysis was designed according to the PRISMA - guidelines for reporting of systematic reviews^42^.

### Quality assessment

The overall quality of the included studies was evaluated by the QUASAD-2 (quality assessment of diagnostic accuracy studies 2) by two different investigators, a tool designed to assess the quality of primary diagnostic accuracy studies, consisting of 4 different domains: patient selection, index test, reference standard, and flow and timing.

### Statistical analysis

EGFR-T790M mutational status in tumor tissue was considered the “gold standard”. For each study, we tabulated the number of true positives (T790M mutation detected both in liquid biopsy and in tumor tissue), false positives (T790M mutation detected in liquid biopsy but not detected in tumor tissue), false negatives (T790M mutation detected in tumor tissue but not detected in liquid biopsy), and true negatives (T790M mutation neither detected in tumor tissue nor detected in liquid biopsy). These numbers of patients were used to calculate sensitivity, specificity, positive predictive value (PPV), negative predictive value (NPV), positive likelihood ratio (PLR), negative likelihood ratio (NLR), diagnostic odds ratio (DOR), and corresponding 95% confidence intervals (95% CI). The PLR is calculated as: sensitivity/(1 − specificity), and represents the likelihood that a positive liquid biopsy for the T790M mutation result would be expected in a patient with T790M detected in tumor tissue, compared to the likelihood that that same result would be expected in a patient without T790M detected in tumor tissue. The NLR is calculated as (1 − sensitivity)/specificity, and represents the likelihood that a negative liquid biopsy for the T790M mutation result would be expected in a patient with T790M detected in tumor tissue, compared to the likelihood that that same result would be expected in a patient without T790M detected in tumor tissue. The *DOR* is a single measure of diagnostic test performance that combines both likelihood ratios, and is calculated as PLR/NLR; it expresses how much greater the odds of having EGFR T790M mutation detected in tumor tissue are for the people with a positive liquid biopsy than for the people with a negative liquid biopsy. Sensitivity, specificity, positive predictive value and negative predictive value were pooled as weighted averages in which the weight of each study is its sample size. Likelihood ratios and diagnostic odds ratios were pooled by the DerSimonian Laird method (random effects model) to incorporate variation among studies. In addition, we generated a summary receiver operating characteristics (sROC) curve and calculated the area under the curve (AUC) of the sROC. Sub-group analyses were performed for sample size (dividing studies in smaller than 60 and larger than 60 patients) and detection methods (real-time PCR (RT-PCR), digital-PCR (dPCR), next-generation sequencing (NGS)). The Spearman correlation between the logit of sensitivity and logit of 1-specificity was calculated to determine the effect of threshold, and a P value < 0.05 indicated significant threshold effect. The heterogeneity (variation in study outcomes between studies) caused by non-threshold effect was measured by the Q test, which is calculated as the weighted sum of squared differences between individual study effects and the pooled effect across studies, and the inconsistency index (I^2^), which describes the percentage of variation across studies that is due to heterogeneity rather than chance. A P-value < 0.05 and a I^2^ value > 50% indicated significant heterogeneity. Meta-regression analysis was performed to identify the source of heterogeneity. As regards the risk of bias across studies, we performed a publication bias analysis using the visual inspection of the Funnel Plot and the Egger’s test. A P-value < 0.05 indicated significant publication bias. Publication bias analyses was performed by MetaEssentials software^36^. All other statistical analyses were performed using the Meta-DiSc software (version 1.4)^36^.

## References

[1] Mok TS (2009). Gefitinib or carboplatin-paclitaxel in pulmonary adenocarcinoma. N Engl J Med.

[2] Rosell R (2012). Erlotinib versus standard chemotherapy as first-line treatment for European patients with advanced EGFR mutation-positive non-small-cell lung cancer (EURTAC): a multicentre, open-label, randomised phase 3 trial. Lancet Oncol.

[3] Mitsudomi T (2010). Gefitinib versus cisplatin plus docetaxel in patients with non-small-cell lung cancer harbouring mutations of the epidermal growth factor receptor (WJTOG3405): an open label, randomised phase 3 trial. Lancet Oncol.

[4] Maemondo M (2010). Gefitinib or chemotherapy for non-small-cell lung cancer with mutated EGFR. N Engl J Med.

[5] Zhou C (2011). Erlotinib versus chemotherapy as first-line treatment for patients with advanced EGFR mutation-positive non-small-cell lung cancer (OPTIMAL, CTONG-0802): a multicentre, open-label, randomised, phase 3 study. Lancet Oncol.

[6] Sequist LV (2013). Phase III study of afatinib or cisplatin plus pemetrexed in patients with metastatic lung adenocarcinoma with EGFR mutations. J Clin Oncol.

[7] Wu YL (2014). Afatinib versus cisplatin plus gemcitabine for first-line treatment of Asian patients with advanced non-small-cell lung cancer harbouring EGFR mutations (LUX-Lung 6): an open-label, randomised phase 3 trial. Lancet Oncol.

[8] Yang JC (2015). Afatinib versus cisplatin-based chemotherapy for EGFR mutation-positive lung adenocarcinoma (LUX-Lung 3 and LUX-Lung 6): analysis of overall survival data from two randomised, phase 3 trials. Lancet Oncol.

[9] Novello S (2016). Metastatic non-small-cell lung cancer: ESMO Clinical Practice Guidelines for diagnosis, treatment and follow-up. Ann Oncol.

[10] Rolfo C (2014). Novel therapeutic strategies for patients with NSCLC that do not respond to treatment with EGFR inhibitors. Cancer Treat Rev.

[11] Suda, K., Rivard, C. J., Mitsudomi, T. & Hirsch, F. R. Overcoming resistance to EGFR tyrosine kinase inhibitors in lung cancer, focusing on non-T790M mechanisms. *Expert Rev Anticancer Ther*, 1–8 (2017). 10.1080/14737140.2017.1355243 28701107

[12] Bollinger, M. K., Agnew, A. S. & Mascara, G. P. Osimertinib: A third-generation tyrosine kinase inhibitor for treatment of epidermal growth factor receptor-mutated non-small cell lung cancer with the acquired Thr790Met mutation. *J Oncol Pharm Pract*, 1078155217712401 (2017). 10.1177/1078155217712401 28565936

[13] T. S. Mok *et al*. Osimertinib or Platinum-Pemetrexed in EGFR T790M-Positive Lung Cancer. *N Engl J Med* (2016). 10.1056/NEJMoa1612674 PMC6762027 27959700

[14] Luo J, Shen L, Zheng D (2014). Diagnostic value of circulating free DNA for the detection of EGFR mutation status in NSCLC: a systematic review and meta-analysis. Sci Rep.

[15] Qiu M (2015). Circulating tumor DNA is effective for the detection of EGFR mutation in non-small cell lung cancer: a meta-analysis. Cancer Epidemiol Biomarkers Prev.

[16] Qian X (2016). Circulating cell-free DNA has a high degree of specificity to detect exon 19 deletions and the single-point substitution mutation L858R in non-small cell lung cancer. Oncotarget.

[17] Oxnard GR (2016). Association Between Plasma Genotyping and Outcomes of Treatment With Osimertinib (AZD9291) in Advanced Non-Small-Cell Lung Cancer. J Clin Oncol.

[18] Ishii H (2015). Digital PCR analysis of plasma cell-free DNA for non-invasive detection of drug resistance mechanisms in EGFR mutant NSCLC: Correlation with paired tumor samples. Oncotarget.

[19] Thress KS (2015). EGFR mutation detection in ctDNA from NSCLC patient plasma: A cross-platform comparison of leading technologies to support the clinical development of AZD9291. Lung Cancer.

[20] Karlovich C (2016). Assessment of EGFR Mutation Status in Matched Plasma and Tumor Tissue of NSCLC Patients from a Phase I Study of Rociletinib (CO-1686). Clin Cancer Res.

[21] Reckamp KL (2016). A Highly Sensitive and Quantitative Test Platform for Detection of NSCLC EGFR Mutations in Urine and Plasma. J Thorac Oncol.

[22] Sacher AG (2016). Prospective Validation of Rapid Plasma Genotyping for the Detection of EGFR and KRAS Mutations in Advanced Lung Cancer. JAMA Oncol.

[23] Sundaresan TK (2016). Detection of T790M, the Acquired Resistance EGFR Mutation, by Tumor Biopsy versus Noninvasive Blood-Based Analyses. Clin Cancer Res.

[24] Takahama T (2016). Detection of the T790M mutation of EGFR in plasma of advanced non-small cell lung cancer patients with acquired resistance to tyrosine kinase inhibitors (West Japan oncology group 8014LTR study). Oncotarget.

[25] Paweletz CP (2016). Bias-Corrected Targeted Next-Generation Sequencing for Rapid, Multiplexed Detection of Actionable Alterations in Cell-Free DNA from Advanced Lung Cancer Patients. Clin Cancer Res.

[26] Seki Y (2016). Picoliter-Droplet Digital Polymerase Chain Reaction-Based Analysis of Cell-Free Plasma DNA to Assess EGFR Mutations in Lung Adenocarcinoma That Confer Resistance to Tyrosine-Kinase Inhibitors. Oncologist.

[27] Thompson JC (2016). Detection of Therapeutically Targetable Driver and Resistance Mutations in Lung Cancer Patients by Next-Generation Sequencing of Cell-Free Circulating Tumor DNA. Clin Cancer Res.

[28] Suzawa K (2017). Optimal method for quantitative detection of plasma EGFR T790M mutation using droplet digital PCR system. Oncol Rep.

[29] Jenkins S (2017). Plasma ctDNA Analysis for Detection of the EGFR T790M Mutation in Patients with Advanced Non-Small Cell Lung Cancer. J Thorac Oncol.

[30] Wang W, Song Z, Zhang Y, Comparison A (2017). of ddPCR and ARMS for detecting EGFR T790M status in ctDNA from advanced NSCLC patients with acquired EGFR-TKI resistance. Cancer Med.

[31] Mellert H (2017). Development and Clinical Utility of a Blood-Based Test Service for the Rapid Identification of Actionable Mutations in Non-Small Cell Lung Carcinoma. J Mol Diagn.

[32] Kasahara N (2017). Plasma epidermal growth factor receptor mutation testing with a chip-based digital PCR system in patients with advanced non-small cell lung cancer. Lung Cancer.

[33] Yoshida H (2017). EGFR T790M Detection in Circulating Tumor DNA from Non-small Cell Lung Cancer Patients Using the PNA-LNA Clamp Method. Anticancer Res.

[34] Wu YL (2017). Conventional real-time PCR-based detection of T790M using tumor tissue or blood in patients with EGFR TKI-resistant NSCLC. Onco Targets Ther.

[35] A. Buder *et al*. Cell-free plasma DNA-guided treatment with osimertinib in patients with advanced EGFR-mutated NSCLC. *J Thorac Oncol* (2018). 10.1016/j.jtho.2018.02.014 29505901

[36] Zamora J, Abraira V, Muriel A, Khan K (2006). A. Coomarasamy, Meta-DiSc: a software for meta-analysis of test accuracy data. BMC Med Res Methodol.

[37] Jenkins S (2017). EGFR Mutation Analysis for Prospective Patient Selection in Two Phase II Registration Studies of Osimertinib. J Thorac Oncol.

[38] Chabon JJ (2016). Corrigendum: Circulating tumour DNA profiling reveals heterogeneity of EGFR inhibitor resistance mechanisms in lung cancer patients. Nat Commun.

[39] Swets JA (1988). Measuring the accuracy of diagnostic systems. Science.

[40] Glas AS (2003). The diagnostic odds ratio: a single indicator of test performance. J Clin Epidemiol.

[41] F. Passiglia *et al*. Metastatic site location influences the diagnostic accuracy of ctDNA EGFR- mutation testing in NSCLC patients: a pooled analysis. *Curr Cancer Drug Targets* (2018). 10.2174/1568009618666180308125110 29521235

[42] Moher D (2010). Preferred reporting items for systematic reviews and meta-analyses: the PRISMA statement. Int J Surg.

