# Publisher Correction: The diagnostic accuracy of circulating tumor DNA for the detection of EGFR-T790M mutation in NSCLC: a systematic review and meta-analysis

**DOI:** 10.1038/s41598-018-35524-y

**Published:** 2018-11-19

**Authors:** Francesco Passiglia, Sergio Rizzo, Massimo Di Maio, Antonio Galvano, Giuseppe Badalamenti, Angela Listì, Leonardo Gulotta, Marta Castiglia, Fabio Fulfaro, Viviana Bazan, Antonio Russo

**Affiliations:** 1 Department of Surgical, Oncological and Oral Sciences, Section of Medical Oncology, University of Palermo, Palermo, Italy; 2 Division of Medical Oncology, Umberto I “Ordine Mauriziano” Hospital, Via Magellano 1, Turin, 10028 Italy; 3 Department of Oncology, University of Turin, Turin, Italy; 4 Department of Surgical, Oncological and Stomatological Disciplines, University of Palermo, Palermo, Italy

Correction to: *Scientific Reports* 10.1038/s41598-018-30780-4, published online 06 September 2018

The original version of this Article contained an error in the order of author names, which was incorrectly given as ‘Francesco Passiglia, Sergio Rizzo, Massimo Di Maio, Antonio Galvano, Giuseppe Badalamenti, Angela Listì, Leonardo Gulotta, Marta Castiglia, Viviana Bazan, Antonio Russo & Fabio Fulfaro’.

In addition, in the original version of this Article the author Massimo Di Maio was incorrectly indexed.

These errors have now been corrected in the HTML and PDF versions of this Article.

